# 2-Acetyl-3,5,5,9-tetra­methyl-6,7,8,9-tetra­hydro-5*H*-benzocyclo­hepten-7-one

**DOI:** 10.1107/S1600536810050610

**Published:** 2010-12-08

**Authors:** Ahmed Benharref, Noureddine Mazoir, Essediya Lassaba, Jean-Claude Daran, Moha Berraho

**Affiliations:** a Laboratoire de Chimie Biomoleculaire, Substances Naturelles et Réactivite’, URAC16, Université Cadi Ayyad, Faculté des Sciences Semlalia, BP 2390, Bd My Abdellah, 40000 Marrakech, Morocco; b Laboratoire de Chimie de Coordination, 205 route de Narbonne, 31077 Toulouse Cedex 04, France

## Abstract

The title compound, C_17_H_22_O_2_, was semi-synthesized from a mixture of α-atlantone (*Z*) and α-atlantone (*E*), which were isolated from the essential oil of the Atlas cedar (*cedrus atlantica*). The mol­ecule consists of fused six- and seven-membered rings. The seven-membered ring is in a screw-boat conformation.

## Related literature

For the isolation of α-atlantone (*Z*) and its isomer α-atlantone (*E*), see: Plattier & Teisseire (1974 ▶). For the reactivity of these ketones, see: Loughzail *et al.* (2009 ▶); Mazoir *et al.* (2009 ▶). For the isolation and reactivity of aryl-himachalene, see: Son Bredenberg & Erdtman (1961 ▶); Daunis *et al.* (1981 ▶) For puckerint parameters, see: Cremer & Pople (1975 ▶).
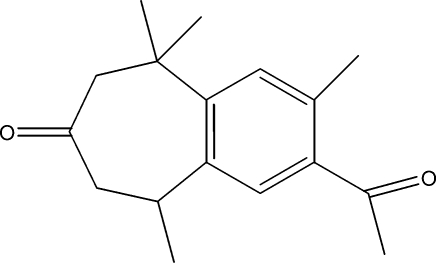


## Experimental

### 

#### Crystal data

C_17_H_22_O_2_
*M* *_r_* = 258.35
Monoclinic, 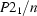

*a* = 7.7996 (6) Å
*b* = 18.3702 (10) Å
*c* = 9.9357 (6) Å
β = 99.616 (7)°
*V* = 1403.59 (16) Å^3^
*Z* = 4
Mo *K*α radiation
μ = 0.08 mm^−1^
*T* = 180 K
0.6 × 0.25 × 0.10 mm

#### Data collection

Oxford Diffraction Xcalibur Eos Gemini ultra diffractometer
14554 measured reflections
2855 independent reflections
2196 reflections with *I* > 2σ(*I*)
*R*_int_ = 0.054

#### Refinement

*R*[*F* ^2^ > 2σ(*F* ^2^)] = 0.052
*wR*(*F* ^2^) = 0.145
*S* = 1.08
2855 reflections
177 parameters
H-atom parameters constrained
Δρ_max_ = 0.61 e Å^−3^
Δρ_min_ = −0.29 e Å^−3^

### 

Data collection: *CrysAlis PRO* (Oxford Diffraction, 2010 ▶); cell refinement: *CrysAlis PRO*; data reduction: *CrysAlis PRO*; program(s) used to solve structure: *SHELXS97* (Sheldrick, 2008 ▶); program(s) used to refine structure: *SHELXL97* (Sheldrick, 2008 ▶); molecular graphics: *ORTEP-3 for Windows* (Farrugia, 1997 ▶); software used to prepare material for publication: *WinGX* (Farrugia, 1999 ▶).

## Supplementary Material

Crystal structure: contains datablocks I, global. DOI: 10.1107/S1600536810050610/lh5174sup1.cif

Structure factors: contains datablocks I. DOI: 10.1107/S1600536810050610/lh5174Isup2.hkl

Additional supplementary materials:  crystallographic information; 3D view; checkCIF report

## Figures and Tables

**Fig. 1.**
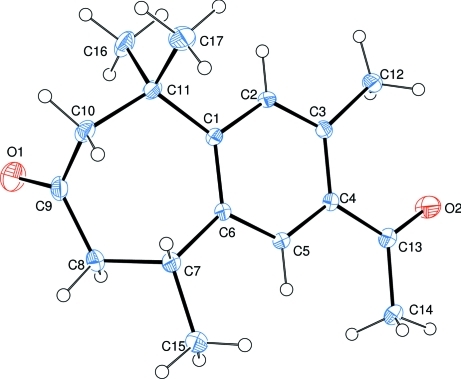
Molecular structure of the title compound with the atom-labelling scheme. Displacement ellipsoids are drawn at the 30% probability level. H atoms are represented as small spheres of arbitrary radii.

## supplementary crystallographic information

### Comment

α-Atlantone (*Z*) and α-atlantone (E) are the two isomeric sesquiterpene ketones which are constituents of the essential oil of Cedrus atlantica (3%) (Plattier & Teisseire 1974). The reactivity of these ketones has been studied by our team (Loughzail *et al.*, 2009; Mazoir *et al.*, 2009) in order to prepare products with high added value used in the cosmetics industry or in pharmacology. In the same context, we have synthesized the title compound (4-acethyl-arylhimachal-9-one) from a mixture of two isomers α- atlatones. The action of one equivalent of chloride cethyl in the presence of the Lewis acid AlCl_3_ on 2-methyl-6-(4-methylphenyl)hept-2-en-4-one, which was obtained from the mixture of two α- atlantones isomers (Mazoir *et al.*, 2009) led to a yield of 35% at 4-acethyl-aryl-himachal-9-one, a derivative of the aryl-himachalene (Son Bredenberg & Erdtman, 1961; Daunis *et al.*, 1981). The structure of this new derivative of aryl-himachalene was determined by ^1^H, ^13^C NMR spectral analysis and mass spectroscopy and confirmed by its single-crystal X-ray structure. The molecular structure of the title compound is shown in Fig.1. The benzene ring is essentially planar, whereas the seven-membered ring displays a screw boat conformation as indicated by Cremer & Pople (1975) puckering parameters QT = 0.9688 (2) Å and θ = 71.57 (10)°, φ2 = 168.10 (11)° and φ3 = -6.36 (4)°.

### Experimental

In a reactor equipped with a stirring stick, containing 2 g (9,30 mmol) of 2-methyl-6-(4-methylphenyl) hept-2-en-4-one; 1,2 g of Lewis acid (AlCl_3_) and 30 ml of dichloromethane, we added drop wise with vigorous stirring 1 ml of acetyl chloride. The reaction mixture is heated to 323K in a water bath for one hour. After cooling, the reaction mixture was poured into 20 ml of iced water supplemented with 4 ml of concentrated hydrochloric acid. The reaction mixture was extracted three times with 20 ml of dichloromethane. The organic phases are combined, dried and evaporated under vacuum. Chromatography on silica gel of the residue obtained with hexane-ethyl acetate (98/2) as eluent, allowed us to isolate the pure 4-acethyl-aryl-himachal-9-one. The title compound was recrystallized in hexane.

### Refinement

All H atoms were fixed geometrically and treated as riding with C—H = 0.96 Å (methyl), 0.97 Å (methylene), 0.93Å (aromatic), 0.98Å (methine) with *U*_iso_(H) = 1.2U_eq_(C) or *U*_iso_(H) = 1.5U_eq_(methyl).

### Crystal data

**Table:**

C_17_H_22_O_2_	*F*(000) = 560
*M**_r_* = 258.35	*D*_x_ = 1.223 Mg m^−^^3^
Monoclinic, *P*2_1_/*n*	Mo *K*α radiation, λ = 0.71073 Å
Hall symbol: -P 2yn	Cell parameters from 2855 reflections
*a* = 7.7996 (6) Å	θ = 3.5–29.3°
*b* = 18.3702 (10) Å	µ = 0.08 mm^−^^1^
*c* = 9.9357 (6) Å	*T* = 180 K
β = 99.616 (7)°	Needle, colourless
*V* = 1403.59 (16) Å^3^	0.6 × 0.25 × 0.10 mm
*Z* = 4	

### Data collection

**Table:**

Oxford Diffraction Xcalibur Eos Gemini ultra diffractometer	2196 reflections with *I* > 2σ(*I*)
Radiation source: fine-focus sealed tube	*R*_int_ = 0.054
graphite	θ_max_ = 26.4°, θ_min_ = 3.5°
Detector resolution: 16.1978 pixels mm^-1^	*h* = −9→9
φ and ω scans	*k* = −22→22
14554 measured reflections	*l* = −12→13
2855 independent reflections	

### Refinement

**Table:**

Refinement on *F*^2^	Primary atom site location: structure-invariant direct methods
Least-squares matrix: full	Secondary atom site location: difference Fourier map
*R*[*F*^2^ > 2σ(*F*^2^)] = 0.052	Hydrogen site location: inferred from neighbouring sites
*wR*(*F*^2^) = 0.145	H-atom parameters constrained
*S* = 1.08	*w* = 1/[σ^2^(*F*_o_^2^) + (0.0729*P*)^2^ + 0.4506*P*] where *P* = (*F*_o_^2^ + 2*F*_c_^2^)/3
2855 reflections	(Δ/σ)_max_ < 0.001
177 parameters	Δρ_max_ = 0.61 e Å^−^^3^
0 restraints	Δρ_min_ = −0.29 e Å^−^^3^

### Special details

**Table:**

Geometry. All e.s.d.'s (except the e.s.d. in the dihedral angle between two l.s. planes) are estimated using the full covariance matrix. The cell e.s.d.'s are taken into account individually in the estimation of e.s.d.'s in distances, angles and torsion angles; correlations between e.s.d.'s in cell parameters are only used when they are defined by crystal symmetry. An approximate (isotropic) treatment of cell e.s.d.'s is used for estimating e.s.d.'s involving l.s. planes.
Refinement. Refinement of *F*^2^ against ALL reflections. The weighted *R*-factor *wR* and goodness of fit *S* are based on *F*^2^, conventional *R*-factors *R* are based on *F*, with *F* set to zero for negative *F*^2^. The threshold expression of *F*^2^ > σ(*F*^2^) is used only for calculating *R*-factors(gt) *etc*. and is not relevant to the choice of reflections for refinement. *R*-factors based on *F*^2^ are statistically about twice as large as those based on *F*, and *R*- factors based on ALL data will be even larger.

### Fractional atomic coordinates and isotropic or equivalent isotropic displacement parameters (Å^2^)

**Table:**

	*x*	*y*	*z*	*U*_iso_*/*U*_eq_	
C1	0.3403 (2)	0.13187 (7)	0.19877 (15)	0.0192 (3)	
C3	0.3247 (2)	0.09640 (8)	−0.04316 (15)	0.0205 (3)	
C4	0.2676 (2)	0.02663 (8)	−0.01250 (15)	0.0204 (3)	
C5	0.2523 (2)	0.01136 (8)	0.12259 (16)	0.0230 (3)	
H5	0.2154	−0.0350	0.1424	0.028*	
C6	0.2887 (2)	0.06101 (8)	0.23016 (15)	0.0217 (3)	
C2	0.3584 (2)	0.14604 (8)	0.06319 (15)	0.0207 (3)	
H2	0.3958	0.1922	0.0430	0.025*	
C13	0.2253 (2)	−0.03137 (9)	−0.11877 (16)	0.0261 (4)	
C14	0.1717 (2)	−0.10595 (9)	−0.07708 (18)	0.0298 (4)	
H14C	0.1449	−0.1363	−0.1565	0.045*	
H14B	0.0710	−0.1017	−0.0338	0.045*	
H14A	0.2653	−0.1273	−0.0145	0.045*	
C10	0.3481 (2)	0.18511 (9)	0.44504 (16)	0.0279 (4)	
H10A	0.4307	0.1503	0.4920	0.034*	
H10B	0.3653	0.2309	0.4940	0.034*	
C11	0.3860 (2)	0.19632 (8)	0.29825 (16)	0.0244 (4)	
C7	0.2791 (2)	0.03531 (9)	0.37574 (16)	0.0279 (4)	
H7	0.3916	0.0462	0.4322	0.033*	
C8	0.1391 (2)	0.07785 (9)	0.43676 (17)	0.0294 (4)	
H13A	0.0269	0.0690	0.3805	0.035*	
H13B	0.1342	0.0586	0.5270	0.035*	
C12	0.3533 (3)	0.12064 (9)	−0.18269 (17)	0.0296 (4)	
H12C	0.4006	0.1690	−0.1770	0.044*	
H12B	0.2445	0.1203	−0.2443	0.044*	
H12A	0.4332	0.0880	−0.2156	0.044*	
C9	0.1675 (2)	0.15840 (9)	0.44834 (17)	0.0304 (4)	
C15	0.2465 (3)	−0.04610 (9)	0.38933 (18)	0.0344 (4)	
H15B	0.1345	−0.0583	0.3384	0.052*	
H15A	0.2495	−0.0581	0.4838	0.052*	
H15C	0.3349	−0.0731	0.3544	0.052*	
C16	0.2815 (3)	0.26390 (9)	0.23925 (19)	0.0398 (5)	
H16A	0.3121	0.2761	0.1523	0.060*	
H16B	0.3081	0.3042	0.3008	0.060*	
H16C	0.1593	0.2534	0.2280	0.060*	
C17	0.5811 (3)	0.21235 (11)	0.3114 (2)	0.0401 (5)	
H17A	0.6462	0.1694	0.3420	0.060*	
H17B	0.6121	0.2509	0.3762	0.060*	
H17C	0.6072	0.2268	0.2242	0.060*	
O1	0.0495 (2)	0.19936 (8)	0.45978 (19)	0.0569 (5)	
O2	0.2338 (2)	−0.01978 (7)	−0.23812 (13)	0.0451 (4)	

### Atomic displacement parameters (Å^2^)

**Table:**

	*U*^11^	*U*^22^	*U*^33^	*U*^12^	*U*^13^	*U*^23^
C1	0.0204 (8)	0.0162 (6)	0.0213 (7)	0.0013 (6)	0.0047 (6)	−0.0012 (5)
C3	0.0206 (8)	0.0216 (7)	0.0201 (7)	0.0008 (6)	0.0053 (6)	0.0008 (6)
C4	0.0216 (8)	0.0197 (7)	0.0206 (7)	0.0009 (6)	0.0056 (6)	−0.0023 (6)
C5	0.0305 (9)	0.0162 (7)	0.0246 (8)	−0.0016 (6)	0.0110 (6)	−0.0008 (6)
C6	0.0284 (8)	0.0181 (7)	0.0207 (7)	−0.0003 (6)	0.0100 (6)	−0.0007 (6)
C2	0.0233 (8)	0.0158 (7)	0.0240 (8)	−0.0002 (6)	0.0065 (6)	0.0019 (5)
C13	0.0299 (9)	0.0236 (7)	0.0255 (8)	0.0000 (6)	0.0065 (7)	−0.0034 (6)
C14	0.0380 (10)	0.0216 (8)	0.0309 (9)	−0.0030 (7)	0.0088 (8)	−0.0075 (6)
C10	0.0407 (10)	0.0212 (7)	0.0209 (8)	0.0002 (7)	0.0023 (7)	−0.0041 (6)
C11	0.0336 (9)	0.0166 (7)	0.0231 (8)	−0.0007 (6)	0.0056 (7)	−0.0023 (6)
C7	0.0406 (10)	0.0226 (7)	0.0227 (8)	−0.0006 (7)	0.0118 (7)	−0.0004 (6)
C8	0.0383 (10)	0.0308 (9)	0.0215 (8)	−0.0040 (7)	0.0118 (7)	−0.0031 (6)
C12	0.0415 (10)	0.0265 (8)	0.0225 (8)	−0.0031 (7)	0.0104 (7)	0.0019 (6)
C9	0.0420 (11)	0.0297 (8)	0.0218 (8)	0.0067 (7)	0.0115 (7)	−0.0022 (6)
C15	0.0477 (11)	0.0278 (8)	0.0305 (9)	0.0021 (8)	0.0150 (8)	0.0045 (7)
C16	0.0705 (14)	0.0206 (8)	0.0293 (9)	0.0125 (8)	0.0109 (9)	−0.0009 (7)
C17	0.0399 (11)	0.0412 (10)	0.0392 (10)	−0.0167 (9)	0.0065 (8)	−0.0103 (8)
O1	0.0600 (10)	0.0397 (8)	0.0797 (12)	0.0175 (7)	0.0368 (9)	−0.0021 (7)
O2	0.0778 (11)	0.0364 (7)	0.0217 (6)	−0.0148 (7)	0.0099 (6)	−0.0065 (5)

### Geometric parameters (Å, °)

**Table:**

C1—C2	1.402 (2)	C11—C17	1.534 (3)
C1—C6	1.412 (2)	C11—C16	1.546 (2)
C1—C11	1.545 (2)	C7—C15	1.527 (2)
C3—C2	1.387 (2)	C7—C8	1.547 (2)
C3—C4	1.407 (2)	C7—H7	0.9800
C3—C12	1.508 (2)	C8—C9	1.498 (2)
C4—C5	1.396 (2)	C8—H13A	0.9700
C4—C13	1.498 (2)	C8—H13B	0.9700
C5—C6	1.398 (2)	C12—H12C	0.9600
C5—H5	0.9300	C12—H12B	0.9600
C6—C7	1.535 (2)	C12—H12A	0.9600
C2—H2	0.9300	C9—O1	1.209 (2)
C13—O2	1.217 (2)	C15—H15B	0.9600
C13—C14	1.510 (2)	C15—H15A	0.9600
C14—H14C	0.9600	C15—H15C	0.9600
C14—H14B	0.9600	C16—H16A	0.9600
C14—H14A	0.9600	C16—H16B	0.9600
C10—C9	1.497 (3)	C16—H16C	0.9600
C10—C11	1.549 (2)	C17—H17A	0.9600
C10—H10A	0.9700	C17—H17B	0.9600
C10—H10B	0.9700	C17—H17C	0.9600
			
C2—C1—C6	117.47 (13)	C15—C7—C6	114.81 (13)
C2—C1—C11	115.01 (12)	C15—C7—C8	108.73 (14)
C6—C1—C11	127.49 (13)	C6—C7—C8	111.27 (13)
C2—C3—C4	117.42 (13)	C15—C7—H7	107.2
C2—C3—C12	117.90 (14)	C6—C7—H7	107.2
C4—C3—C12	124.68 (14)	C8—C7—H7	107.2
C5—C4—C3	118.18 (13)	C9—C8—C7	115.09 (14)
C5—C4—C13	119.40 (13)	C9—C8—H13A	108.5
C3—C4—C13	122.41 (13)	C7—C8—H13A	108.5
C4—C5—C6	124.41 (14)	C9—C8—H13B	108.5
C4—C5—H5	117.8	C7—C8—H13B	108.5
C6—C5—H5	117.8	H13A—C8—H13B	107.5
C5—C6—C1	117.48 (13)	C3—C12—H12C	109.5
C5—C6—C7	118.96 (13)	C3—C12—H12B	109.5
C1—C6—C7	123.50 (13)	H12C—C12—H12B	109.5
C3—C2—C1	124.97 (14)	C3—C12—H12A	109.5
C3—C2—H2	117.5	H12C—C12—H12A	109.5
C1—C2—H2	117.5	H12B—C12—H12A	109.5
O2—C13—C4	121.39 (15)	O1—C9—C10	122.13 (16)
O2—C13—C14	119.23 (14)	O1—C9—C8	121.14 (18)
C4—C13—C14	119.37 (14)	C10—C9—C8	116.73 (15)
C13—C14—H14C	109.5	C7—C15—H15B	109.5
C13—C14—H14B	109.5	C7—C15—H15A	109.5
H14C—C14—H14B	109.5	H15B—C15—H15A	109.5
C13—C14—H14A	109.5	C7—C15—H15C	109.5
H14C—C14—H14A	109.5	H15B—C15—H15C	109.5
H14B—C14—H14A	109.5	H15A—C15—H15C	109.5
C9—C10—C11	113.10 (14)	C11—C16—H16A	109.5
C9—C10—H10A	109.0	C11—C16—H16B	109.5
C11—C10—H10A	109.0	H16A—C16—H16B	109.5
C9—C10—H10B	109.0	C11—C16—H16C	109.5
C11—C10—H10B	109.0	H16A—C16—H16C	109.5
H10A—C10—H10B	107.8	H16B—C16—H16C	109.5
C17—C11—C1	108.73 (13)	C11—C17—H17A	109.5
C17—C11—C16	109.32 (15)	C11—C17—H17B	109.5
C1—C11—C16	108.79 (14)	H17A—C17—H17B	109.5
C17—C11—C10	106.74 (14)	C11—C17—H17C	109.5
C1—C11—C10	116.12 (12)	H17A—C17—H17C	109.5
C16—C11—C10	106.99 (13)	H17B—C17—H17C	109.5
